# Differences in colonic crypt morphology of spontaneous and colitis-associated murine models via second harmonic generation imaging to quantify colon cancer development

**DOI:** 10.1186/s12885-019-5639-8

**Published:** 2019-05-09

**Authors:** Sandra P. Prieto, Cassandra L. Reed, Haley M. James, Kyle P. Quinn, Timothy J. Muldoon

**Affiliations:** Biomedical Engineering Department, University of Arkansas, Fayetteville, AR 72701 USA

**Keywords:** Colorectal cancer, Inflammatory bowel disease, Multiphoton, Second harmonic generation, Image analysis, Crypt morphology, Azoxymethane, Dextran sulfate sodium salt

## Abstract

**Background:**

Colorectal cancer remains the second leading cause of cancer death in the United States, and increased risk in patients with ulcerative colitis (a subset of inflammatory bowel disease) has motivated studies into early markers of dysplasia. The development of clinically translatable multiphoton imaging systems has allowed for the potential of in vivo label-free imaging of epithelial crypt structures via autofluorescence and/or second harmonic generation (SHG). SHG has been used to investigate collagen structures in various types of cancer, though the changes that colorectal epithelial collagen structures undergo during tumor development, specifically colitis-associated tumors, have not been fully investigated.

**Methods:**

This study used two murine models, using A/J mice, one for spontaneous carcinoma and one for colitis-associated carcinoma, to investigate and quantify SHG image features that could potentially inform future study designs of endoscopic multiphoton imaging systems. The spontaneous tumor model comprised a series of six weekly injections of azoxymethane (AOM model). The colitis-associated tumor model comprised a single injection of AOM, followed by cycles of drinking water with dissolved dextran sodium sulfate salt (AOM-DSS model). SHG images of freshly resected murine colon were acquired with a multiphoton imaging system, and image features, such as crypt size, shape and distribution, were quantified using an automated algorithm.

**Results:**

The comparison of quantified features of crypt morphology demonstrated the ability of our quantitative image feature algorithms to detect differences between spontaneous (AOM model) and colitis-associated (AOM-DSS model) murine colorectal tissue specimens. There were statistically significant differences in the mean and standard deviation of nearest neighbor (distance between crypts) and circularity between the Control cohort, AOM and AOM-DSS cohorts. We also saw significance between AOM and AOM-DSS cohorts when calculating nearest neighbor in images acquired at fixed depths.

**Conclusion:**

The results provide insight into the ability of SHG imaging to yield relevant data about the crypt microstructure in colorectal epithelium, specifically the potential to distinguish between spontaneous and colitis-associated murine models using quantification of crypt shape and distribution, informing future design of translational multiphoton imaging systems and protocols.

**Electronic supplementary material:**

The online version of this article (10.1186/s12885-019-5639-8) contains supplementary material, which is available to authorized users.

## Background

Inflammatory bowel disease (IBD) is characterized by chronic inflammation of the gastrointestinal tract and is manifested clinically as either ulcerative colitis (UC) or Crohn’s disease [1]. While there is currently no specific histological feature that is used to definitively distinguish between UC and Crohn’s disease, an important distinction is the distribution pattern of the inflammation. UC is characterized by severe inflammation beginning at the rectum and continuing throughout the colon; Crohn’s disease is characterized by lesions over Peyer’s patches, and non-continuous regions of inflammation that span the entire depth of the intestinal wall [2–4].

Patients with UC exhibit an increased risk for colorectal cancer (CRC); approximately 29% of UC patients develop CRC within 10 years of diagnosis, while 2.9% with Crohn’s disease develop CRC after 10 years of disease [5–8]. There are different types of lesions that may be detected in a UC patient during colonoscopy: two types associated with intraepithelial neoplasia are a dysplasia-associated lesion/mass (DALM) and an adenoma-like mass (ALM) [9, 10]. Flat or raised lesions without sharp contrast (delineation) against non-dysplastic epithelium are typically classified as DALMs, and are frequently associated with malignancy [10]. ALMs have features similar to sporadic adenomas that are observed in non-IBD patients [11]. Preliminary studies have suggested that standard polypectomy, a complete endoscopic resection of the ALM mass, followed by endoscopic surveillance is highly successful in patients with an ALM and could spare the patient a colectomy, since of the 34 patients in one study only one patient developed an adenocarcinoma following the initial polypectomy [9–11]. Unfortunately, in 50–80% of DALM cases the lesions are not visible during standard endoscopy [10, 12].

Several modalities of endoscopic imaging have emerged to improve detection of flat or discolored lesions [13]. Among them, multiphoton microendoscopy uses nonlinear optical processes to yield label-free high-resolution image data and rapid three-dimensional image acquisition up to several hundred microns in depth without the need for tissue resection and subsequent fixation [14]. These systems have recently overcome a number of specific technical challenges, including obtaining uniform scans, low sensitivity, durability and reliability of the scanner, and miniaturization of the distal scan mechanism [15, 16]. Miniaturization of multiphoton laser microscopy systems greatly enhances the clinical translatability of this modality, leading towards in vivo imaging applications in gastrointestinal epithelial tissues [17–19]. A form of multiphoton imaging, second harmonic generation (SHG) is a nonlinear optical process whereby non-centrosymmetric crystalline structures (such as collagen) interact with two low energy photons nearly simultaneously, resulting in the generation of a new photon at twice the energy (or half the wavelength of the incident photons). Multiphoton endoscopic systems capable of SHG imaging show great potential in improving clinical decision-making for patients with IBD. In the colon, collagen structures can be used to quantify changes in the three-dimensional crypt microstructure in the setting of IBD and associated dysplastic transformation. A systematic, quantitative model of these optical biomarkers of dysplasia progression could guide clinical translation of SHG imaging approaches for IBD patients.

Our study was designed to further develop this biomarker model via SHG imaging and quantification of morphological changes that occur in the crypt structures of murine models of spontaneous and colitis-associated colorectal tumors. A series of azoxymethane (AOM) injections was used as a murine model for spontaneous (ALM-like) tumor progression as it induces polypoid growth in the distal colon, similar to human CRC [20]. AOM injections followed by subsequent administration of dextran sodium sulfate salt (DSS) are considered to have long-term effects that produce cancerous flat lesions or dysplasia-associated lesion or mass (DALM-like) similar to those observed in humans [21]. We also acquired SHG images of epithelial tissue over discrete timepoints to investigate if temporal changes in crypt microstructure could be detected and quantified at varying stages of tumor progression. Key SHG image features could serve as optical biomarkers; a translatable measure towards detection of lesions and distinguishing ALM and DALM type lesions endoscopically in patients with known IBD.

## Methods

### Murine models and colorectal tissue procurement

**Fig. 1:**
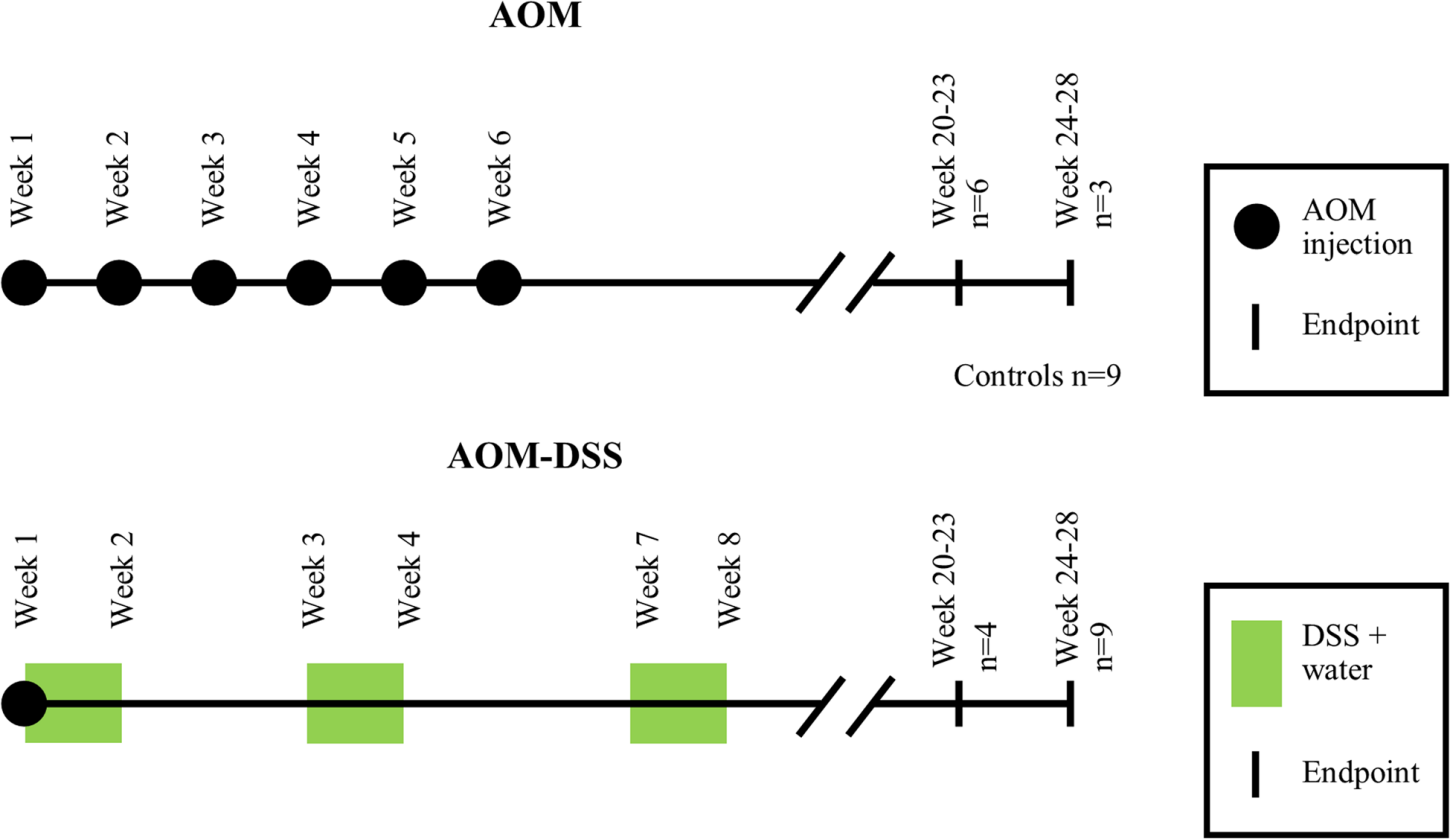
Timeline of murine models. Week 1 begins at the first azoxymethane injection, for mice at 10 weeks of age. Number of mice euthanized per time point and cohort labelled by n values

Two murine models were used for this study: a spontaneous colorectal carcinoma model and a colitis-induced colorectal carcinoma model [20]. Thirty-one *A/J* mice (The Jackson Laboratory, ME, USA) were used in accordance with the University of Arkansas Institutional Animal Care and Use Committee-approved protocol #18093. Mice were received at 9 weeks of age and allowed to acclimate for one week in a temperature and light controlled facility; mice were maintained on an 12 h day-night cycle, with a bedding mixture of approximately 75% aspen chip bedding (7090A, Envigo, USA) and 25% paper product bedding (7099, Envigo, USA). All procedures were conducted in the light phase. Food (Tekland 8640, Envigo, USA) and water were provided ad libitum (Fig. 1). Mice were anesthetized using 1.5% isoflurane (IsoThesia, Henry Schein Animal Health, OH, USA) and oxygen (Compressed USP Oxygen, Airgas, PA, USA) from a precision vaporizer (911,103, VetEquip, CA, USA), then placed on a heating pad (TP700, Stryker Medical, MI, USA) with a nosecone for constant supply of 1.5% isoflurane.

The spontaneous colorectal carcinoma model (referred to as the AOM model), consisted of a series of six weekly subcutaneous injections of AOM (A5486, Sigma-Aldrich, Inc., MO, USA) diluted in sterile saline, at a dose of 10 mg per kg body weight [20]. Control mice underwent a series of six weekly subcutaneous injections of saline (injection site over the shoulders). AOM and Control cohorts acclimated and were injected in parallel. The colitis-induced colorectal carcinoma model (referred to as the AOM-DSS model) consisted of a single AOM injection (at 10 mg/kg body weight, subcutaneous administration), followed by several courses of drinking water supplemented with DSS (36,000–50,000 molecular weight, SKU 0216011080, MP Biomedicals, OH, USA) at a concentration of 1.5% (w/v) (Fig. 1) [20]. AOM-DSS cohorts acclimated and were injected a few months after the AOM and Control cohorts. Subcutaneous AOM injections were administered with 28 gauge syringe needles (BD329410, VWR, PA, USA) in volumes of less than 200 μL. For the AOM-DSS model, beginning on the day of the AOM injection, mice were provided free access to DSS solution for seven days [20]. On Day 8, the mice were provided untreated drinking water ad libitum for 14 days, and then the DSS-solution and untreated water cycle was repeated at Day 22 and Day 53 (Fig. 1) [20].

Post treatment, mice were provided food and water ad libitum and were weighed and inspected daily until two weeks after concluding AOM/DSS treatment, after which they were inspected daily and weighed weekly. Mice were euthanized at early (20–23 weeks) or late (24–28 weeks) time points, representative of early or late-stage carcinoma progression (Fig. 1). Mice within each cohort were randomly selected for euthanizing at either early or late timepoints. Figure 1 notes the number of mice per cohort: Control *n* = 9, AOM early *n* = 6, AOM late *n* = 3, AOM-DSS early *n* = 4, AOM-DSS late *n* = 9.

AOM-injected mice develop lesions and small polyps at approximately 20 weeks, with tumors progressing in size over the subsequent weeks; mice began to show signs of rectal bleeding past 28 weeks and were therefore euthanized [20]. Mice were euthanized via anesthetized cervical dislocation; administered anesthetic was as previously described (1.5% isoflurane and oxygen). Murine colons were resected within 15 min of euthanasia, and a 1 cm section of distal colon was longitudinally sectioned and placed on a coverslip for imaging. An adjacent section of tissue was fixed in formalin and paraffin-embedded for reference H&E staining.

### Multiphoton microscopy imaging system

The microscopy system comprises a titanium-sapphire ultrafast femtosecond pulsed laser source (Mai Tai eHP, Spectra-Physics, CA, USA), and galvo-resonant scan head scanners which acquires a fixed 15 frames per second (MPM-SCAN64J, Thorlabs, USA). The laser power was controlled via a quarter-wave plate and a polarizing beam splitter, to reduce the incoming excitation power at the sample to the range of tens to hundreds of milliwatts. The light was then circularly polarized, to reduce orientation bias of SHG in collagen, by use of a half-wave plate and a second polarizing beam splitter. Circular polarization was determined by measuring intensity of orthogonal collagen fibers at multiple regions, and selecting the optimal positioning of the half-wave plate for reduction of intensity variation at different orientations. Following the scanning optics, the illumination laser beam (at 800 nm wavelength) passed through the back aperture of a 20x water immersion objective (0.85 NA, Nikon, NY, USA), prior to illuminating the sample, which was placed above the objective on an inverted microscopy stage. Backscattered SHG signal was collected via the objected and traveled through a 635 nm long pass dichroic beamsplitter (Di02-R635, Semrock, NY, USA), a 447/60 nm filter set (447/60 nm 25 mm dia. Filter, Semrock, NY, USA), a 400 nm/40 nm filter set (400/40 nm 25 mm dia. Filter, Semrock, NY, USA), and photomultiplier tube (H7422 PASO, Hamamatsu, Japan). The entire optical system resides inside an enclosure to reduce ambient light.

### Second harmonic generation image acquisition

**Fig. 2:**
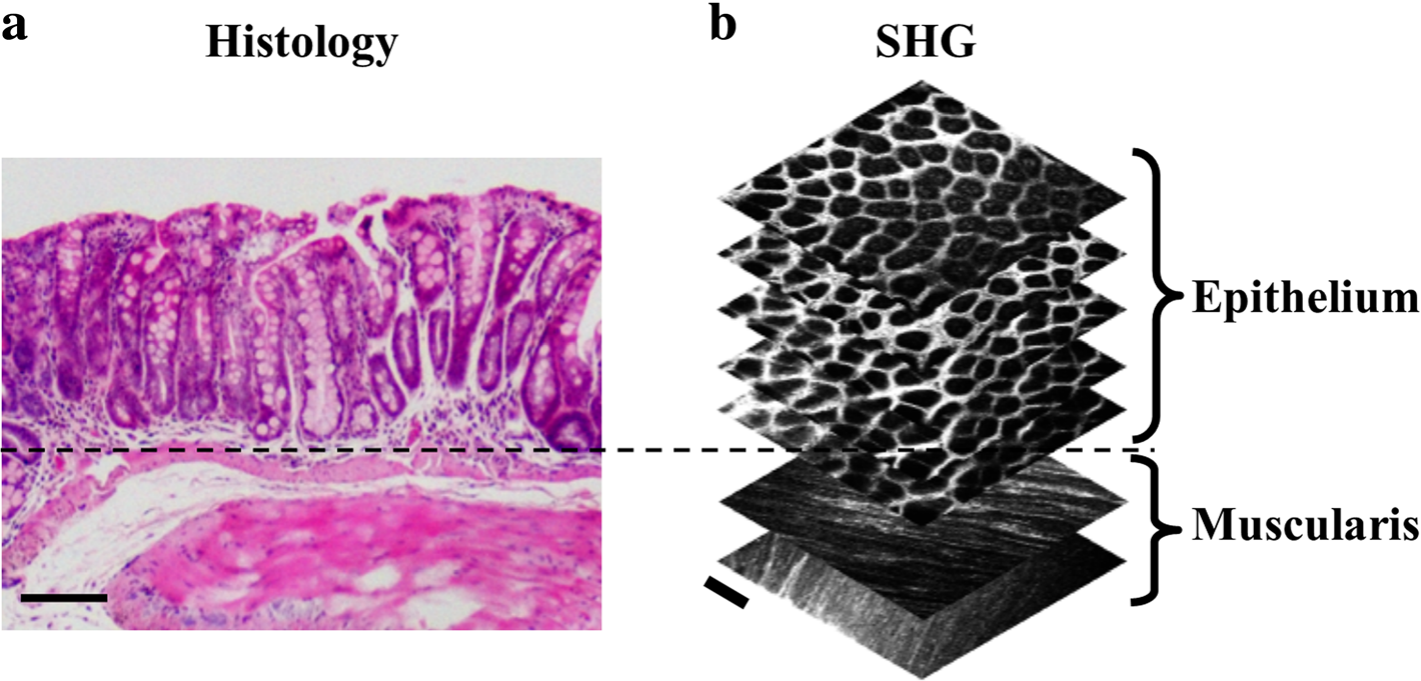
Comparison of histology and SHG images of Control murine colonic epithelium. (**a**) H&E stained transverse section of Control murine colonic epithelium; scale bar is 100 μm. (**b**) Example of an SHG stack shown, with images of depths at 20 μm, 40 μm, 60 μm, 80 μm, and 100 μm (epithelial layer), and 160 μm and 180 μm (muscularis layer), from top to bottom. Scale bar is 100 μm

Freshly resected tissue was placed epithelium-down on a 24 × 40 mm No. 1 coverslip, then imaged using our non-linear optical microscopy imaging system described above. Total image acquisition time was approximately 1.5 s, with total optical power at the sample limited to prevent photo-damage. Individual images were acquired at 512 × 512 pixels at 14-bit depth, yielding a 522 μm × 522 μm (~ 0.27 mm^2^) field of view, at approximately 20 μm from the epithelial surface. Image stacks were acquired at consecutive depths from the epithelial surface, in 20 μm steps, from 20 μm to 100 μm below the epithelial surface (Fig. 2).

### Optimization of automated crypt detection sensitivity

In previous work, we developed an algorithm for determining optimal parameters for binarizing grayscale images of colon epithelium [22, 23]. The image intensities were re-scaled between 0 and 1, and the thresholds for binarizing were optimized for maximizing the ability of the algorithm to segment crypts can vary depending on the strength of the SHG signal in the tissue (details are included in Additional file 1: Supplemental Information). The selected optimal parameters for the SHG image data set was calculated to have a 67% crypt detection sensitivity (CDS), when compared to manual selection of crypt locations.

### Quantification of crypt image segmentation

**Fig. 3:**
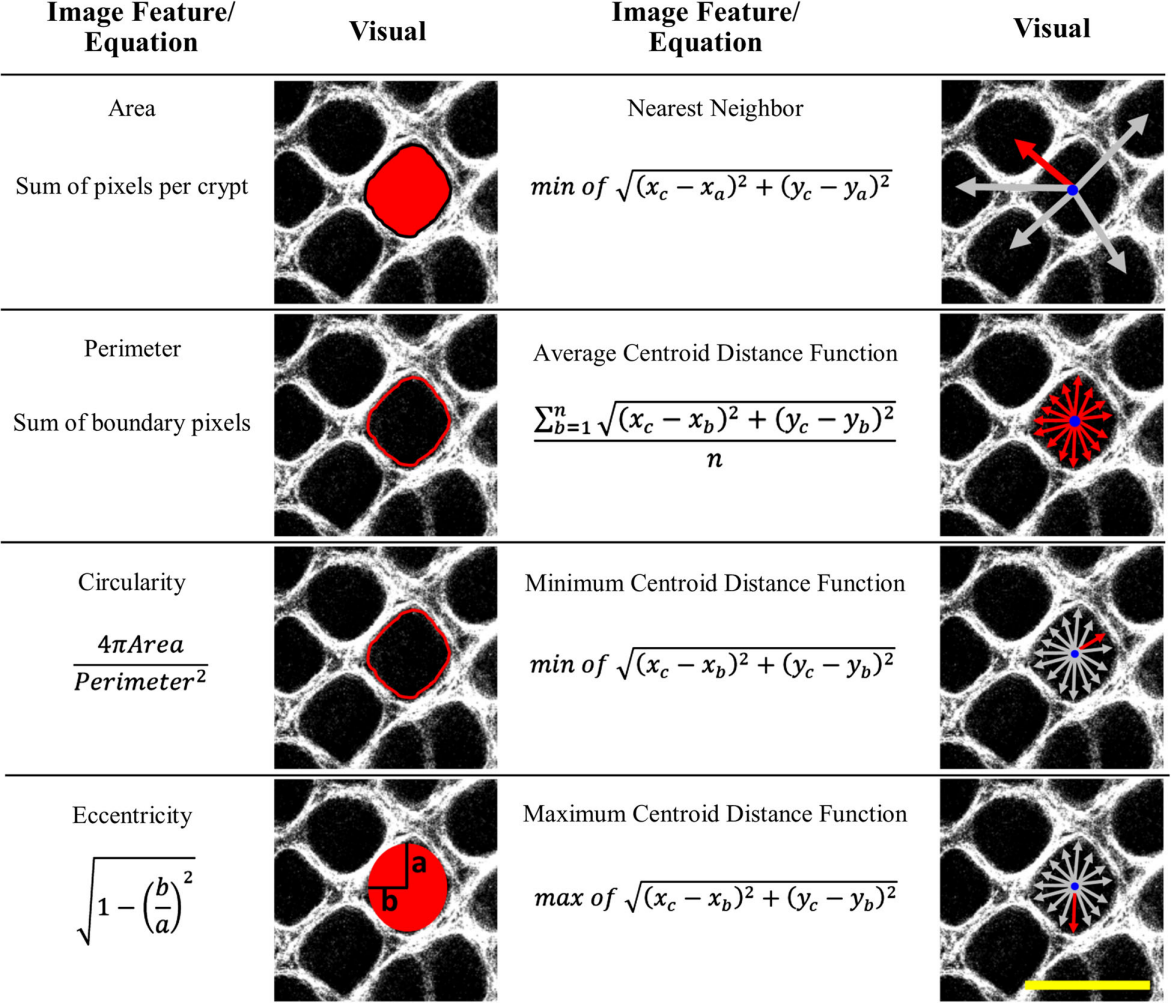
Summary of quantitative image features. Each image feature is described with an equation and a visual representation of the calculation using an SHG image of normal epithelium; scale bar is 100 μm. For eccentricity, a represents half the length of the major axis, and b represents half the length of the minor axis. For nearest neighbor, (x_c_,y_c_) represents the centroid coordinate and (x_a_,y_a_) represent the centroid coordinates of adjacent crypts; the arrows represent the distance to adjacent crypts, the red arrow represents the distance selected as the nearest neighbor. For the centroid distance functions (CDF), (x_c_,y_c_) represents the centroid coordinate and (x_b_,y_b_) represent the boundary pixel coordinates. The average CDF was calculated by averaging all the distances to the boundary pixels (red arrows); the minimum CDF was calculated by selecting the distance to the closest boundary pixel (red arrow); the maximum CDF was calculated by selecting the distance to the furthest boundary pixel (red arrow)

Following the optimization of the threshold combinations for the algorithm’s % CDS, the optimized algorithm was applied to the image set and the images were converted into a binary image, where white objects were defined as the detected crypt structures [22, 23]. Figure 3 summarizes the image features that were quantified, as well provides a visual representation of the calculation. Details of the image segmentation algorithm are included in Additional file 1: Supplemental Information. Early stages of CRC typically include regions of abnormal morphology, for example, aberrant crypt foci (ACF) which are enlarged or non-tubular (branching) crypts, and/or multiple layers of cells lining a crypt [24, 25]. We chose to measure various crypt morphology image features related to changes viewed during histopathology, including crypt area, circularity, and distribution. The mean and standard deviation (referred to as variance in the results) of each image feature within each image were compared via a nested one-way ANOVA. The standard deviation of the image features within each image was calculated in order to measure the heterogeneity of the crypt structures; for example, tumor-adjacent regions tend to have both abnormally large and abnormally small crypt structures, which could produce a mean in a normal range, but the standard deviation of crypt areas within an image would retain the heterogeneous nature.

The results of the quantification of image features were then compared across cohorts: AOM early time point, AOM late time point, AOM-DSS early time point, AOM-DSS late time point, and Control. Nested one-way ANOVA with Tukey’s post-test was used to determine statistically significant differences between the cohorts (JMP Pro 13).

### Depth section image analysis

Each sample was imaged from 20 μm to 100 μm below the epithelial surface in 20 μm increments (Fig. 2); depth stacks were acquired from each sample at one to three locations on the tissue section. Regions in normal epithelium were selected randomly. Regions in AOM and AOM-DSS epithelium were selected adjacent to tumors; tumors were characterized as epithelial regions with SHG signal but no discernable crypt structures. These depth section images were then analyzed with the algorithm to quantify the previously described image features. The results were then compared across depths, as well as across AOM and AOM-DSS cohorts. Nested two-way ANOVA with Tukey’s post-test was used to determine statistically significant differences between the cohorts (JMP Pro 13).

## Results

### Qualitative comparison of second harmonic generation images

**Fig. 4:**
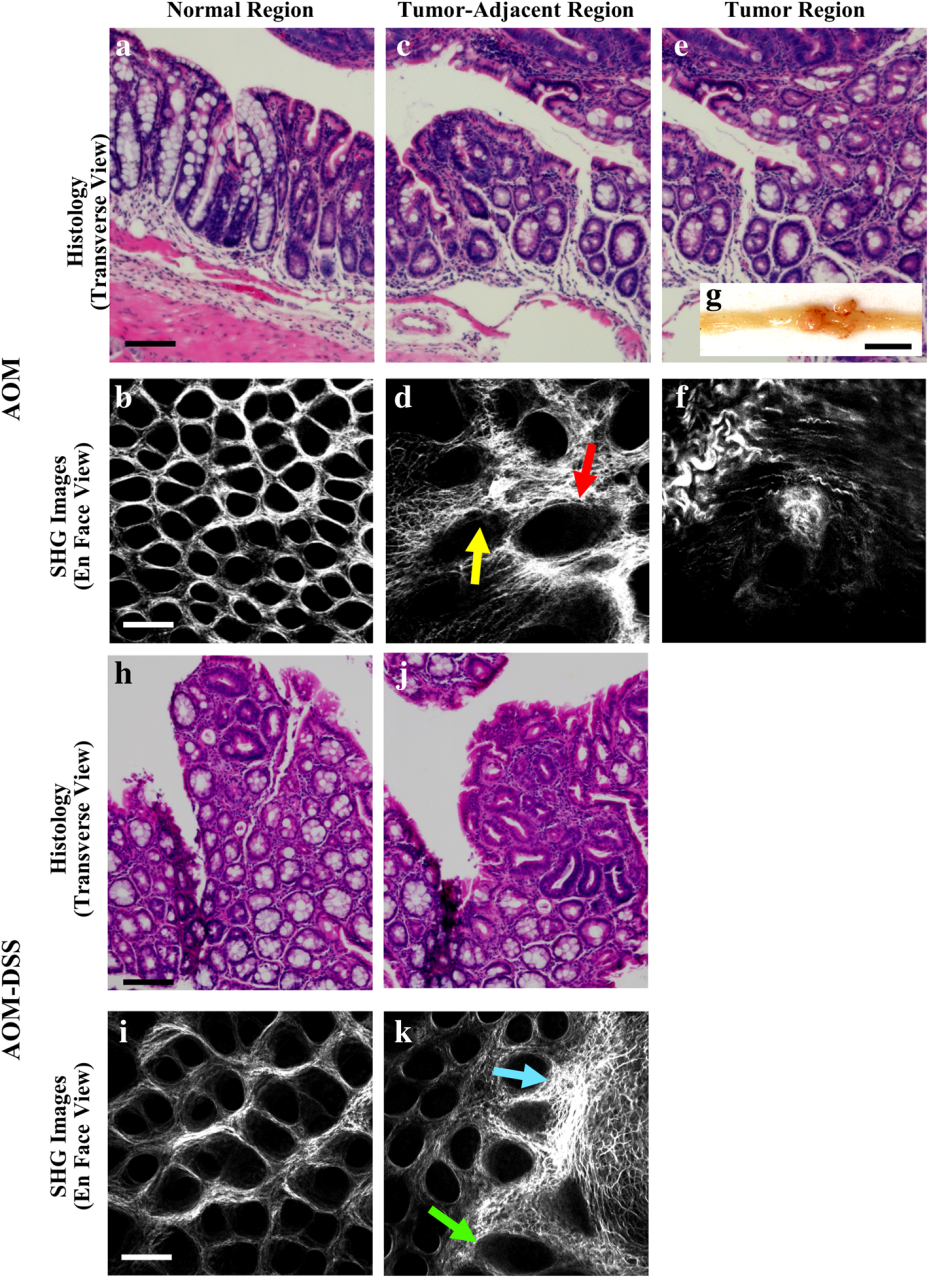
Examples of conventional H&E histology and SHG images of AOM and AOM-DSS murine tissue. (**a**, **b**, **h**, **i**) normal epithelium, (**c**, **d**, **j**, k) epithelium adjacent to a tumor, and (**e**, **f**) tumor region; all scale bars are 100 μm. (**g**) Image of a longitudinally sectioned AOM murine colon, exposing the epithelium and a grossly visible tumor; scale bar is 0.5 cm. (**d**) Red arrow points to an abnormally large crypt; yellow arrow points to an example of a serrated edge. (**k**) green arrow points to an enlarged crypt in the tumor-adjacent region; blue arrow point toward tumor edge. All images were manually contrast enhanced for display

Figure 4 shows examples of histology and SHG of both AOM and AOM-DSS murine models. Normal crypt structures tends to be uniform in size and general shape across the field of view, with crypt shape being tubular in transverse view (Fig. 4a) and roughly circular in *en face* view (Fig. 4b, h, i). Collagen distribution appears relatively even throughout the field of view (Fig. 4b, i). Crypt structures directly adjacent to tumor regions, or in regions of early dysplasia, tend to vary in size across a field of view, with one or more crypts being enlarged (Fig. 4c, d, k), and crypts often being oblong and/or having serrated edges in *en face* view (Fig. 4e). SHG images of grossly visible tumors do not show any discernable crypt structures (Fig. 4f, k).

### Increased mean distance between crypts in AOM vs. AOM-DSS cohorts

**Fig. 5:**
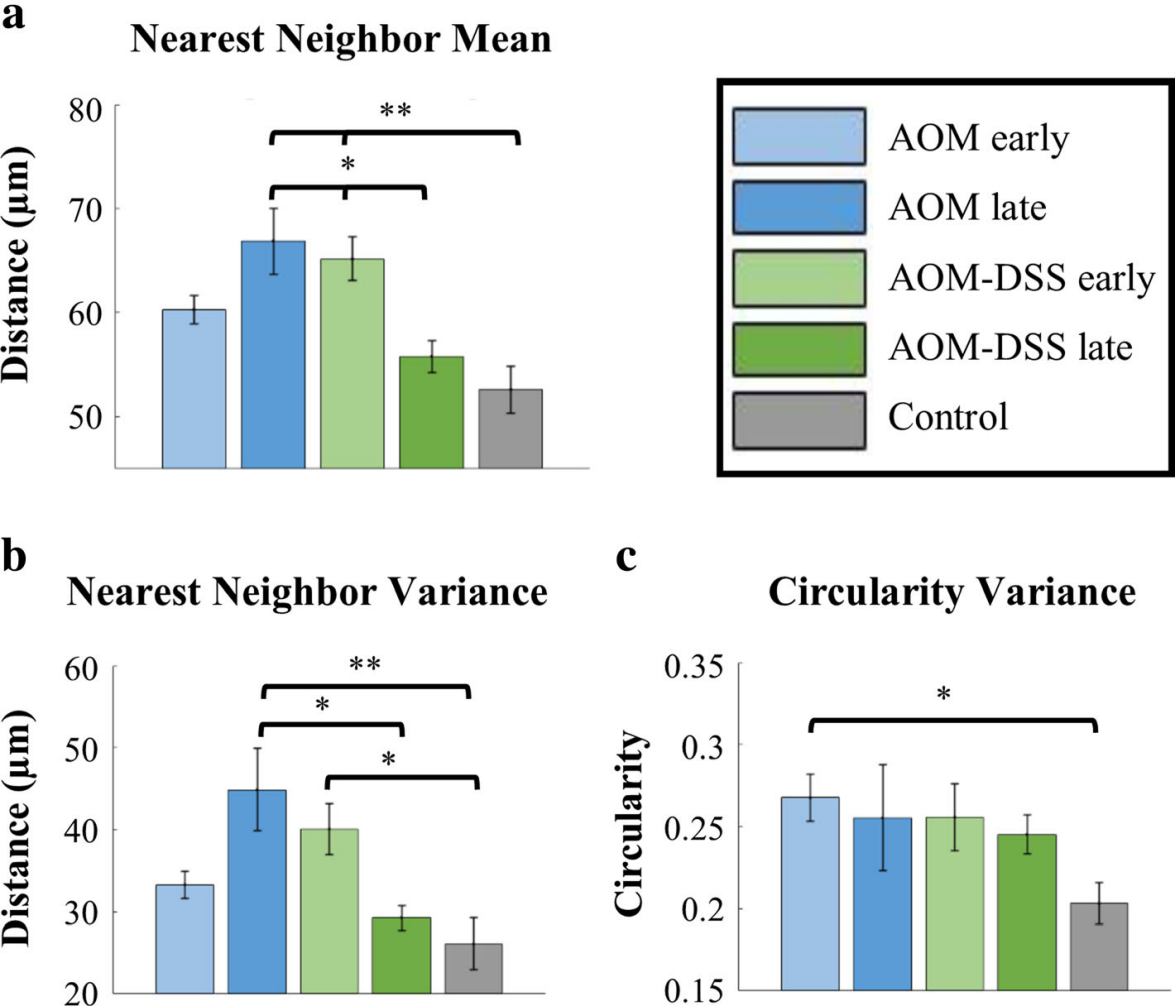
(**a**) Mean values of nearest neighbor, (**b**) the mean values of the standard deviation of crypt nearest neighbor, and (**c**) the mean values of the standard deviation of circularity. Cohort n values (images): AOM early *n* = 48, AOM late *n* = 23, AOM-DSS early *n* = 45, AOM-DSS late *n* = 24, Control *n* = 55. Error bars are standard error; significance was calculated via a nested one-way ANOVA with Tukey’s HSD post-test. Significance was noted (*) for *p* values < 0.05 and (**) for *p* values < 0.01

Nearest neighbor and crypt circularity image features showed significant differences between spontaneous CRC tumor and colitis-associated tumor models. Figure 5 shows statistically significant comparisons of the cohorts for the mean of the nearest neighbor (a), and the standard deviation (variance) of the nearest neighbor (b) and circularity (c). Results for all image features, including non-statistically significant, are included as Additional file 1: Supplemental Figures. There was statistical significance when comparing AOM and AOM-DSS cohorts using nearest neighbor quantification (Fig. 5 a, b). Mean nearest neighbor was greater for AOM late and AOM-DSS early cohorts as compared to the Control (Fig. 5a). AOM late cohort mean nearest neighbor was also greater than the AOM-DSS early cohort (Fig. 5a). When comparing the variance of nearest neighbor, once again AOM late and AOM-DSS early cohorts had greater values than the Control (Fig. 5b). The variance of the AOM late group was also significantly greater than the AOM-DSS late group (Fig. 5 b). Measuring the variance of circularity of the cohorts showed a difference between the AOM early cohort and the Control (Fig. 5c).

### Differences in circularity and mean distance between crypts in tumor-bearing vs. control cohorts at varying acquisition depths

**Fig. 6:**
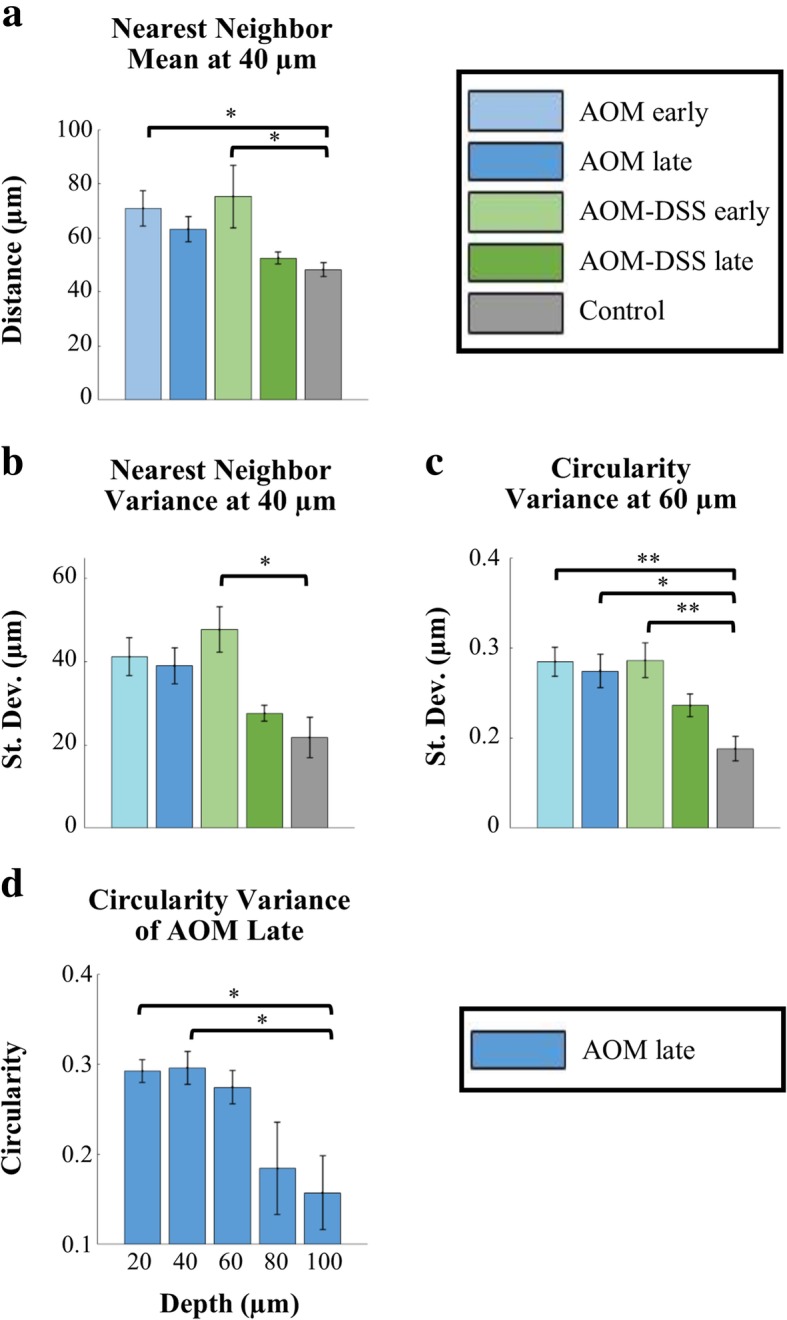
(**a**, **b**) Mean and standard deviation of crypt nearest neighbors at 40 μm depth. (**c**) Standard deviation of crypt circularities at 60 μm depth. (**d**) Standard deviation of crypt circularity at a range of imaging depths in microns, for AOM late cohort. Cohort n values (stacks): AOM early *n* = 8, AOM late *n* = 9, AOM-DSS early n = 5, AOM-DSS late *n* = 12, Control *n* = 9. Significance was calculated via two-way ANOVA with Tukey’s post-test. Error bars are standard error. Significance was noted (*) for *p* values < 0.05 and (**) for *p* values < 0.01

As previously stated, images were taken at increasing depths: 20, 40, 60, 80, and 100 μm into the epithelial layer. We applied the image feature quantification algorithm to each depth, per treatment cohort. There were 43 image stacks (5 images in a stack, 20–100 μm) in total between all 5 cohorts. Mean and standard deviation of image features was compared across cohorts, at specific depths of acquisition (Fig. 6). As previously described, the mean and standard deviation (variance) within an image of each image feature were compared via a nested one-way ANOVA. Results for all image features with at least one statistically significant difference between two cohorts are included as Additional file 1: Supplemental Figures. There were significant differences between the Control cohort and AOM cohorts, and Control and AOM-DSS cohorts, for mean nearest neighbor at depth of specifically 40 μm, as well as standard deviation of circularity at a depth of specifically 60 μm, when comparing image stacks. No image feature showed significant differences between the AOM early and AOM late cohorts, nor the AOM early and AOM-DSS early cohorts (Fig. 6a-c).

### Decreased crypt circularity at increased acquisition depth for AOM late cohort

As previously stated, images were taken at increasing depths: 20, 40, 60, 80, and 100 μm into the epithelial layer. Mean and standard deviation of image features were compared across depth of acquisition within each cohort (Fig. 6d) via a nested one-way ANOVA. Results for all image features with at least one statistically significant difference between two cohorts are included as Additional file 1: Supplemental Figures. There was no significance for mean of image features within cohorts; there was significance within the AOM late cohort for standard deviation of circularity (Fig. 6d). There were differences between 100 μm and the more superficial 20 and 40 μm, for standard deviation of circularity of the AOM late cohort.

## Discussion

Various modalities of endoscopic imaging have emerged with the goal of improving early detection of flat or discolored lesions, such as chromoendoscopy, narrow band imaging, and laser microendoscopy [13, 26]. Chromoendoscopy consists of spraying a colorimetric stain, such as indigo carmine, to provide contrast during colonoscopy [27]. Narrow band imaging has been described as label-free chromoendoscopy, as the optical filters only allow imaging of a small range of wavelengths, which enhance contrast in blood vessels and capillary patterns without the need for a contrast agent [26, 27]. While chromoendoscopy and narrow band imaging have shown promise for improving detection of dysplastic/neoplastic lesions, they were not able to distinguish between the similar structures of ALM and DALM lesions [13]. Laser confocal microendoscopy features high spatial resolution - able to resolve cellular and subcellular structures - providing more detailed imaging features that relate more closely to histopathological features [10]. The Cellvizio® confocal laser microendoscopy system showed potential in differentiating between DALM lesions and adjacent normal colorectal epithelial crypt structure in a preliminary clinical trial [28]. Inserting the Cellvizio® endoscopic probe into an existing working channel during colonoscopy showed that DALM regions had enlarged crypts, increased irregularly in the distribution of crypts, increased space between crypts, as well as crypt destruction or fusion [28]. In contrast to confocal laser microendoscopy, multiphoton microendoscopy has additional benefits, including the ability to acquire label-free images of structural/morphological information (via SHG imaging) as well as insight into metabolic information (via autofluorescence of intrinsic biomolecules, such as nicotinamide adenine dinucleotide and flavin adenine dinucleotide, among others) up to several hundred microns in depth [29–32].

SHG imaging, as shown in this manuscript, is well-suited for characterization of collagen structures, especially collagen type I which makes up much of extracellular matrix and connective tissues [29, 33]. SHG imaging of colorectal epithelium highlights crypt shape, as the collagen fibers provide structure surrounding the cellular arrangement without the need for any exogenous contrast agent [34]. We have described our analysis of colorectal epithelial microstructure from SHG images of freshly resected murine colons using automated quantification of morphological image features. These results - a comparison of quantified features of crypt morphology (especially nearest neighbor and circularity) - demonstrated the ability of our quantitative image feature algorithms to detect differences between spontaneous (AOM model) and colitis-associated (AOM-DSS model) murine colorectal tissue specimens. Limitations of SHG imaging include the ability to acquire structural but not cellular information, as well as the need for clinician/investigator judgement to recognize some additional parameters during imaging, such as the presence of tumors which are not detected nor measured by the automated quantification algorithm.

Future work in this area will include murine models of familial adenomatous polyposis, and other hereditary models, to more thoroughly investigate the morphological features of the most common types of CRC. A common murine model of familial adenomatous polyposis - featuring a deletion of the *APC* gene - develop spontaneous adenomas in the colon, although these lesions may not fully exhibit the physiological features of the disease as seen in humans [35]. Despite some limitations, the *APC/min* mouse model and other models will be included in future SHG imaging studies in order to expand the scope of the current work, which is limited to spontaneous and colitis-associated tumor development.

The image biomarkers in this study relate to crypt morphology features that are analogous to conventional endoscopic biopsy and histopathology: crypt shape, size and spatial distribution. Statistically significant differences between spontaneous tumor (AOM) and colitis-associated tumor (AOM-DSS) cohorts indicate that different models of tumor progression can potentially be quantified via SHG imaging, possibly distinguishing between early and late tumor progression. However, none of the image features - which are based solely on collagen-derived SHG signal - in this study showed statistical differences within tumor-bearing cohorts, or between early and late time points of AOM cohorts. It is possible that combining SHG data with autofluorescence imaging data could be a promising technique for acquiring intraoperative complementary data in order to improve endoscopic differentiation between ALM and DALM lesions in patients with UC, but this would require additional study.

These results provide a quantitative model of the morphological changes that crypts undergo during dysplastic transformation, and can serve to guide future translational investigation and clinical studies in patients with IBD, with the long-term goal of reducing morbidity associated with prophylactic colectomy.

## Conclusion

A number of factors contribute to dysplastic transformation in colon epithelium, and it is important to fully understand disease progression in order to improve techniques and tools for screening, diagnosis, and treatment. While studies of collagen fiber structure using optical imaging in cancers such as cervical have been conducted [17], investigation of collagen structures in murine CRC studies has been understudied [36]. Multiphoton endoscopic imaging has shown to be a feasible technology for clinical translation due to its miniaturization and label-free image acquisition. The introduction of image analysis algorithms into computer-ai diagnostic methods yield complementary data to conventional endoscopic procedures, which could lead to improved clinical decision-making for patients with known IBD. The methods described here provide insight into the ability of SHG imaging to yield relevant data about the crypt microstructure in colorectal epithelium, specifically the potential to distinguish between ALM and DALM murine models using quantification of crypt shape and distribution, informing future design of translational multiphoton imaging systems and protocols.

## Additional file

Additional file 1: **Supplementary Information.** Contains additional details on the optimization of automated crypt detection sensitivity, the method for selecting quantifiable image features, the method for image segmentation and quantification of crypt structures, as well as additional results of image segmentation and quantification of crypt structures and image depth stack results. (DOCX 5593 kb)

## Abbreviations

ALM: adenoma-like mass
AOM: Azoxymethane
CDS: Crypt detection sensitivity
CRC: Colorectal cancer
DALM: Dysplasia-associate lesion/mass
DSS: Dextran sodium sulfate salt
IBD: Inflammatory bowel disease
SHG: Second harmonic generation
UC: Ulcerative colitis

## References

[1] Molodecky NA, Soon IS, Rabi DM, Ghali WA, Ferris M, Chernoff G, Benchimol EI, Panaccione R, Ghosh S, Barkema HW, Kaplan GG (2012). Increasing incidence and prevalence of the inflammatory bowel diseases with time, based on systematic review. Gastroenterology.

[2] Podolsky DK (1991). Inflammatory bowel disease. N Engl J Med.

[3] Hogan WJ, Hensley GT, Geenen JE (1980). Endoscopic evaluation of inflammatory bowel disease. Med Clin North Am.

[4] Goldman H (1996). Significance and detection of dysplasia in chronic colitis. Cancer: Interdiscip Int J Am Cancer Soc.

[5] Karvellas CJ, Fedorak RN, Hanson J, Wong CK (2007). Increased risk of colorectal cancer in ulcerative colitis patients diagnosed after 40 years of age. Canadian J Gastroenterol Hepatol.

[6] Ekbom A, Helmick C, Zack M, Adami H (1990). Ulcerative colitis and colorectal cancer: a population-based study. N Engl J Med.

[7] Greenstein AJ, Sachar DB, Smith H, Janowitz HD, Aufses AH (1981). A comparison of cancer risk in Crohn's disease and ulcerative colitis. Cancer.

[8] Rubin DC, Shaker A, Levin MS (2012). Chronic intestinal inflammation: inflammatory bowel disease and colitis-associated colon cancer. Front Immunol.

[9] Ullman T, Odze R, Farraye FA (2008). Diagnosis and management of dysplasia in patients with ulcerative colitis and Crohn's disease of the colon. Inflamm Bowel Dis.

[10] Neumann H, Vieth M, Langner C, Neurath MF, Mudter J (2011). Cancer risk in IBD: how to diagnose and how to manage DALM and ALM. World J Gastroenterol.

[11] Odze RD, Farraye FA, Hecht JL, Hornick JL (2004). Long-term follow-up after polypectomy treatment for adenoma-like dysplastic lesions in ulcerative colitis. Clin Gastroenterol Hepatol.

[12] Shanahan F, Weinstein W, Bernstein C (1994). Are we telling patients the truth about surveillance colonoscopy in ulcerative colitis?. Lancet.

[13] Kiesslich R, Goetz M, Lammersdorf K, Schneider C, Burg J, Stolte M, Vieth M, Nafe B, Galle PR, Neurath MF (2007). Chromoscopy-guided endomicroscopy increases the diagnostic yield of intraepithelial neoplasia in ulcerative colitis. Gastroenterology.

[14] Campagnola P. Second harmonic generation imaging microscopy: applications to diseases diagnostics. ACS Publications. 2011. 10.1021/ac1032325 PMC3104727 21446646

[15] Huland DM, Brown CM, Howard SS, Ouzounov DG, Pavlova I, Wang K, Rivera DR, Webb WW, Xu C (2012). In vivo imaging of unstained tissues using long gradient index lens multiphoton endoscopic systems. Biomedical Optics Express.

[16] Bao H, Boussioutas A, Jeremy R, Russell S, Gu M (2010). Second harmonic generation imaging via nonlinear endomicroscopy. Opt Express.

[17] Zhang Y, Akins ML, Murari K, Xi J, Li MJ, Luby-Phelps K, Mahendroo M, Li X (2012). A compact fiber-optic SHG scanning endomicroscope and its application to visualize cervical remodeling during pregnancy. Proc Natl Acad Sci U S A.

[18] Helmchen F, Fee MS, Tank DW, Denk W (2001). A miniature head-mounted two-photon microscope: high-resolution brain imaging in freely moving animals. Neuron.

[19] Rivera DR, Brown CM, Ouzounov DG, Pavlova I, Kobat D, Webb WW, Xu C (2011). Compact and flexible raster scanning multiphoton endoscope capable of imaging unstained tissue. Proc Natl Acad Sci U S A.

[20] Neufert C, Becker C, Neurath MF (2007). An inducible mouse model of colon carcinogenesis for the analysis of sporadic and inflammation-driven tumor progression. Nat Protoc.

[21] Araki Y, Mukaisyo K, Sugihara H, Fujiyama Y, Hattori T (2010). Increased apoptosis and decreased proliferation of colonic epithelium in dextran sulfate sodium-induced colitis in mice. Oncol Rep.

[22] Prieto SP, Lai KK, Laryea JA, Mizell JS, Muldoon TJ (2016). Quantitative analysis of ex vivo colorectal epithelium using an automated feature extraction algorithm for microendoscopy image data. J Med Imaging.

[23] Prieto SP, Lai KK, Laryea JA, Mizell JS, Mustain WC, Muldoon TJ (2017). Fluorescein as a topical fluorescent contrast agent for quantitative microendoscopic inspection of colorectal epithelium. Biomedical Optics Express.

[24] Kozlowski C, Jeet S, Beyer J, Guerrero S, Lesch J, Wang X, Devoss J, Diehl L (2013). An entirely automated method to score DSS-induced colitis in mice by digital image analysis of pathology slides. Dis Model Mech.

[25] Takayama T, Katsuki S, Takahashi Y, Ohi M, Nojiri S, Sakamaki S, Kato J, Kogawa K, Miyake H, Niitsu Y (1998). Aberrant crypt foci of the colon as precursors of adenoma and cancer. N Engl J Med.

[26] Machida H, Sano Y, Hamamoto Y, Muto M, Kozu T, Tajiri H, Yoshida S (2004). Narrow-band imaging in the diagnosis of colorectal mucosal lesions: a pilot study. Endoscopy.

[27] Curvers W, Baak L, Kiesslich R, Van Oijen A, Rabenstein T, Ragunath K, Rey JF, Scholten P, Seitz U, Ten Kate F, Fockens P, Bergman J (2008). Chromoendoscopy and narrow-band imaging compared with high-resolution magnification endoscopy in Barrett's esophagus. Gastroenterology.

[28] De Palma GD, Staibano S, Siciliano S, Maione F, Siano M, Esposito D, Persico G (2011). In-vivo characterization of DALM in ulcerative colitis with high-resolution probe-based confocal laser endomicroscopy. World J Gastroenterol.

[29] Roth S, Freund I (1979). Second harmonic generation in collagen. J Chem Phys.

[30] Sepehr R, Staniszewski K, Maleki S, Ranji M, Jacobs ER, Audi S (2012). Optical imaging of tissue mitochondrial redox state in intact rat lungs in two models of pulmonary oxidative stress. J Biomed Opt.

[31] Anonymous *Proceedings of the Multiphoton Microscopy in the Biomedical Sciences XIV:* International Society for Optics and Photonics; 2014.

[32] Zhuo S, Chen J, Wu G, Xie S, Zheng L, Jiang X, Zhu X (2010). Quantitatively linking collagen alteration and epithelial tumor progression by second harmonic generation microscopy. Appl Phys Lett.

[33] Freund I, Deutsch M, Sprecher A (1986). Connective tissue polarity. Optical second-harmonic microscopy, crossed-beam summation, and small-angle scattering in rat-tail tendon. Biophys J.

[34] Zhuo S, Zhu X, Chen J, Xie S, Wu G (2011). Quantitative biomarkers of colonic dysplasia based on intrinsic second-harmonic generation signal. J Biomed Opt.

[35] Moser AR, Pitot HC, Dove WF (1990). A dominant mutation that predisposes to multiple intestinal neoplasia in the mouse. Science.

[36] Wang S, Chen J, Yang Y, Jiang W, Feng C, Guan G, Zhuo S, Chen Z (2015). Assessment of tumor invasion depth in colorectal carcinoma using multiphoton microscopy. IEEE Photonics J.

