# Supplementary material for: Differences in colonic crypt morphology of spontaneous and colitis-associated murine models via second harmonic generation imaging to quantify colon cancer development
Source: BMC Cancer. 2019 May 9;19:428. doi: 10.1186/s12885-019-5639-8 (PMC6507102; doi:10.1186/s12885-019-5639-8)
Supplement: Supplementary file 1 — Supplementary Information. Contains additional details on the optimization of automated crypt detection sensitivity, the method for selecting quantifiable image features, the method for image segmentation and quantification of crypt structures, as well as additional results of image segmentation and quantification of crypt structures and image depth stack results. (DOCX 5593 kb) [file 12885_2019_5639_MOESM1_ESM.docx]

**Supplementary Information**

**S1. Optimization of automated crypt detection sensitivity**

In previous work, we developed an algorithm for determining optimal contrast-enhancing parameters for binarizing grayscale images of colon epithelium [1, 2]. The acquired second harmonic generation (SHG) images were re-scaled between 0 and 1, and the thresholds for binarizing were optimized for maximizing the ability of the algorithm to segment crypts, which can vary depending on the strength of the SHG signal in the tissue. Figure S1 shows an example of how different thresholds are optimal for different images. A range of thresholds were applied to the images, and the percent crypt detection sensitivity (% CDS) of each group was calculated against the manual selection of crypts by the authors, as done in previous work [1]. An example of the % CDS of various thresholds on SHG images of murine colonic tissue from azoxymethane-injected mice is shown in Fig. S1(e). The thresholds were values between 0 and 1, used to determine binarization based on all intensities greater than the threshold being converted to white and all intensities less than the threshold being converted to black. The thresholds we tested are smaller than 0.01 since the raw image intensities are low for back-scattered SHG signal. The threshold with highest overall % CDS was 0.0035 with 38% CDS.

Figure S1, specifically (a-d), also shows an example of how different thresholds are optimal for different images, thus affecting % CDS when a single threshold is chosen. Previous work showed that applying a range of thresholds to the images increased the % CDS of the algorithm [1, 2]. The detected crypts for each threshold were compared to the detected crypts of the previous threshold, and duplicates were excluded while newly-detected crypts were stored. In order to determine the optimal combination of thresholds, the algorithm applied various combinations of thresholds to the image set and calculated the % CDS of each combination. Since thresholds between 0.003 and 0.005 showed % CDS higher than 20%, the range of tested combinations included thresholds between 0.0025 and 0.0055, in 0.0005 incremental steps. Thresholds were tested in combination of two, three, four, and five thresholds. The optimal combination of thresholds was determined to be a combination of three thresholds: 0.003, 0.0035, and 0.0045 was calculated to have a 67% CDS. Combinations of four or more thresholds increased % CDS to almost 69%, but also carry the potential for overtraining the algorithm with our data set, therefore the combination of three thresholds was selected. The selected optimal parameters for the SHG image data set was calculated to have a 67% crypt detection sensitivity (CDS), when compared to manual selection of crypt locations.

**
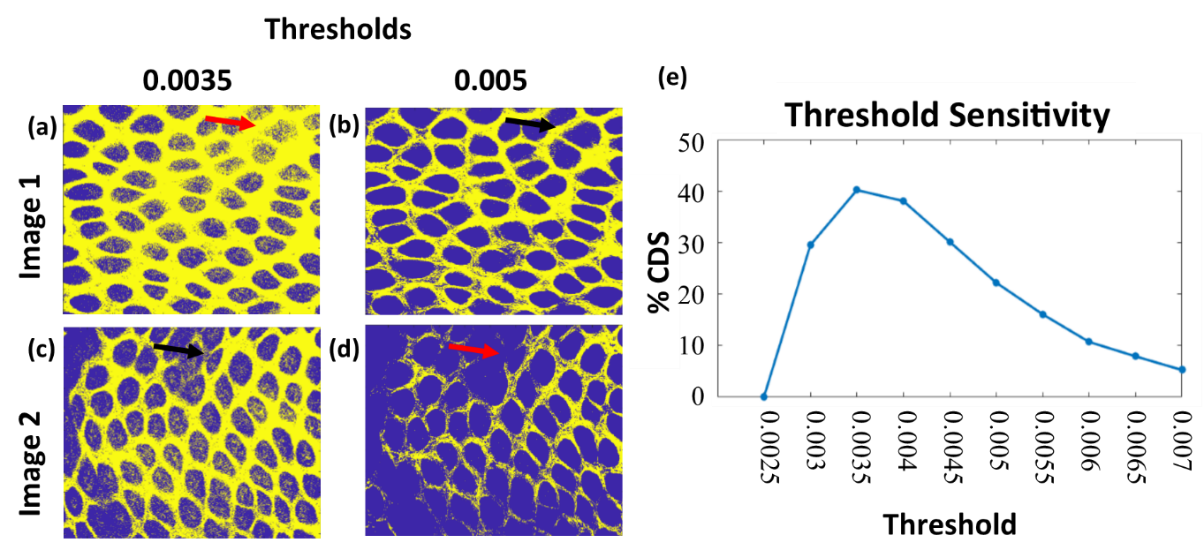
**

**Figure S1.** (a-d) Example of two images from Control mice, binarized using different thresholds. Yellow is SHG signal, blue is background. Red arrows point to crypts with uncertain definition at a given threshold; black arrows point to matching crypts that have clear definition at a different threshold. (e) Plot of percent crypt detection sensitivity (% CDS) of the algorithm at different threshold values.

**S2. Selection of quantifiable image features**

Crypt and cellular morphology are image features used by pathologists when determining a diagnosis for both spontaneous and colitis-associated tumors. Image features include enlarged or non-tubular crypt shapes, and/or crypts with more than a single layer of cells surrounding it. Based on the crypt morphology changes that occur during tumor development, we tested various image morphology parameters for viability as indicators of colon cancer, specifically crypt shape, size, and distribution. We tested various image features in order to provide additional information for investigations using multiphoton endoscopic systems, but we acknowledge that there are limitations to SHG imaging and crypt morphology when analyzed independent of additional factors. For example, Figure S2 shows SHG images of Control tissue, as well as of AOM-DSS early and AOM-DSS late cohorts. Our crypt detection algorithm is designed to detect and analyze crypts, but tumors, such as the ones labelled with yellow arrows, are not recognized during automated analysis. There is still the need for the judgement of a clinician/investigator during the imaging process.

We describe SHG as a complementary optical imaging process to autofluorescence multiphoton imaging. There have been a number of studies on cancer imaging using autofluorescence imaging using endoscopic multiphoton systems [3]. An example of the combined effect of SHG and autofluorescence imaging of a Control tissue sample is shown in Figure S2. Autofluorescence imaging can acquire cellular and subcellular detail, while SHG imaging acquires detail of collagen structures. Autofluorescence in Fig. S2 was acquired using a multiphoton microscopy system (described on page 7 of the main manuscript) which uses a titanium-sapphire ultrafast femtosecond pulsed laser source (Mai Tai eHP, Spectra-Physics, CA, USA) which was set to 750nm excitation, and the signal was collected using a 466/40 bandpass filter (Semrock, NY, USA) and photomultiplier tube (H7422 PASO, Hamamatsu, Japan). We believe that the combination of SHG and autofluorescence imaging in endoscopic multiphoton studies of colorectal cancer could elucidate additional morphological changes and markers of neoplasia and cancer.


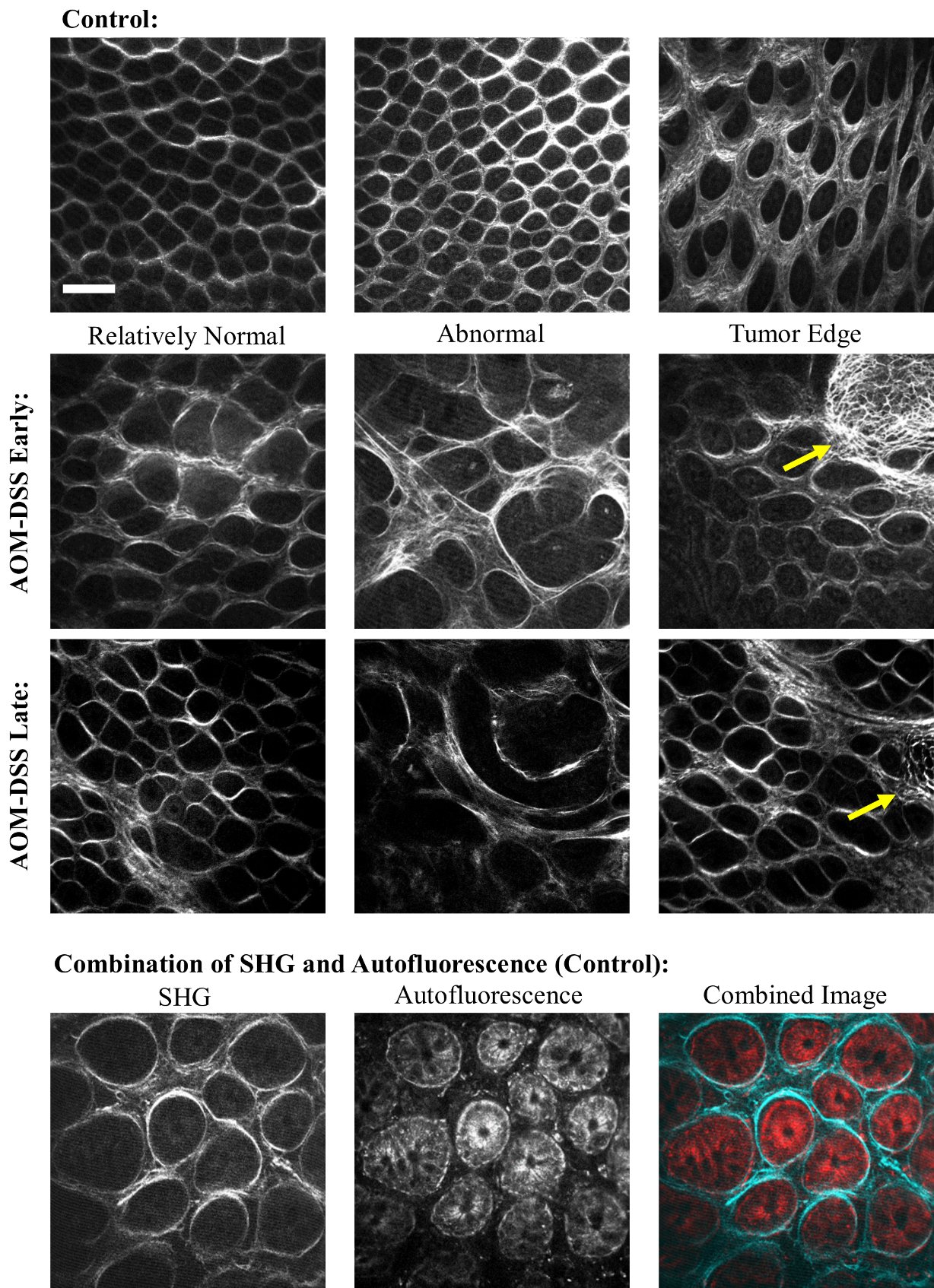


**Figure S2.** Example SHG images of Control, AOM-DSS early, and AOM-DSS late tissue; yellow arrows point to tumor edge. Also, example of the combination of SHG and autofluorescence imaging, using Control epithelium. Scale bar is 100 μm.

**S3. Image segmentation and quantification of crypt structures**

After the selection of the optimal thresholds, the images were binarized, and the objects in the binary image were then eroded and dilated (5 pixel diameter disk) to discard objects too small to be crypts as well as reduce rough edges stemming from image noise.

The segmented objects, or detected crypt structures, were then analyzed for quantification of the following image features: crypt area, circularity, eccentricity, perimeter, distance to the nearest neighbor (distance from each centroid to the closest centroid), and average of a centroid distance function (distance from the centroid to each boundary pixel). Previous work details specifics of automated crypt area and circularity quantification [1, 2]. For automated crypt area, circularity, and perimeter quantification (Fig. S3), the automated quantification of area (A) and perimeter (P), and therefore by default circularity variables, were converted into microns.


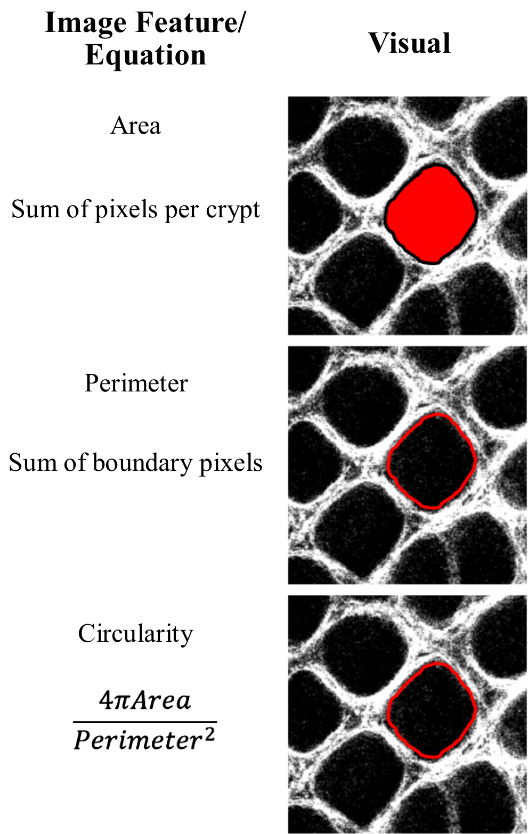


**Figure S3.** Summary of quantitative image features. Each image feature is described with an equation and a visual representation of the calculation using an SHG image of normal epithelium; scale bar is 100 μm.

Additional image features included: eccentricity, where a is half the length of the major axis and b is half the length of the minor axis, and distance to the nearest neighbor, which was calculated by measuring the Euclidean distance between the centroid of a crypt to the centroid of every other crypt in the image, and selecting the minima, where the (x_c_, y_c_) are the coordinates of the centroid and (x_a_, y_aa_) represents the coordinates of all other centroids (Fig. S4). The average centroid distance function was calculated by measuring the Euclidean distance between the centroid of a crypt to each of the boundary pixels, and taking an average of the distances per crypt, where (x_c_, y_c_) are the coordinates of the centroid and (x_b_, y_b_) are the coordinates of a boundary pixel, and *n* is the number of boundary pixels in the object (Fig. S4).


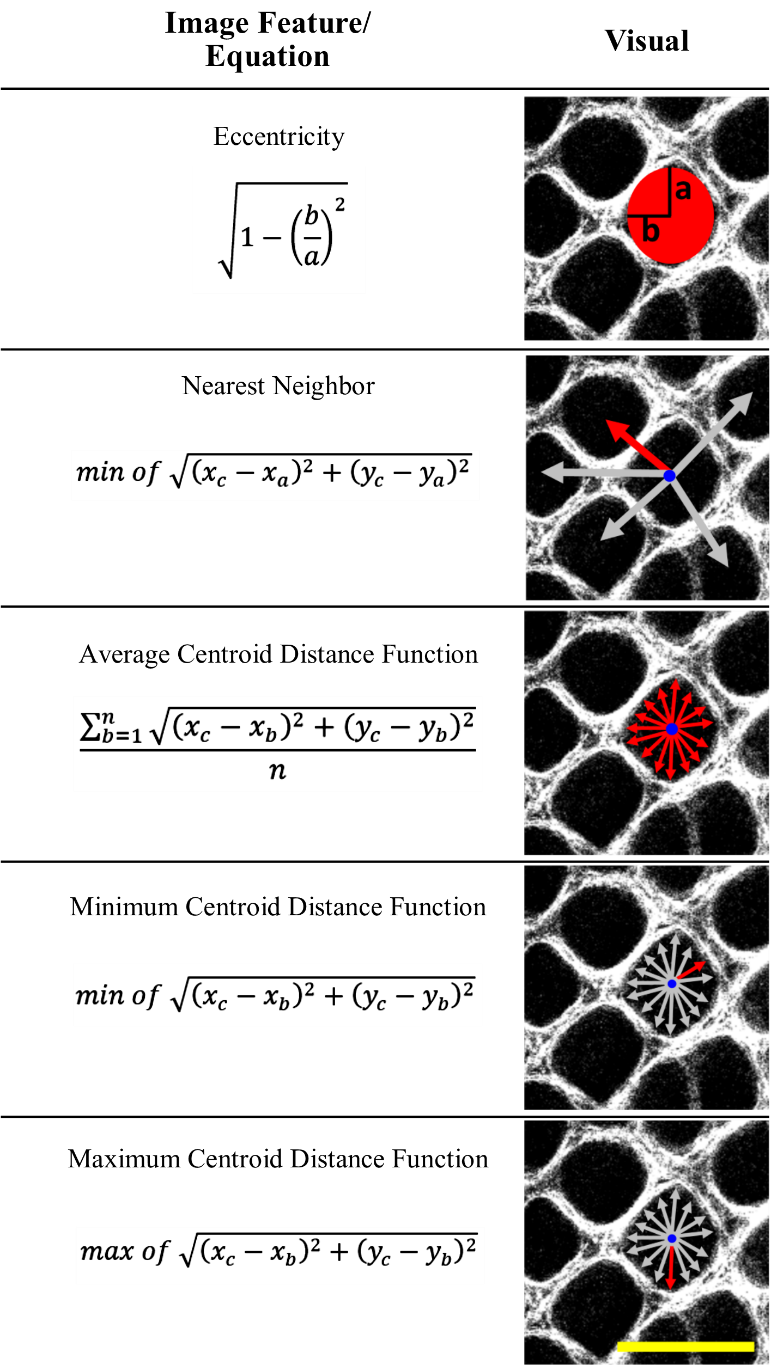


**Figure S4.** Summary of quantitative image features. Each image feature is described with an equation and a visual representation of the calculation using an SHG image of normal epithelium; scale bar is 100 μm.

**S4. Additional results of image segmentation and quantification of crypt structures**

A full set of the results of the one-way ANOVA comparison, including those not shown in the manuscript and those which are not statistically significant, are shown below. In general, for both individual images acquired at a fixed depth (20 μm) and image depth stacks, the mean and standard deviation of each image feature (Fig. S3 and S4) within each image were quantified using an adaptation of a previously developed algorithm [1, 2]. The standard deviation of the image features within each image was calculated in order to measure the heterogeneity of the crypt structures; for example, tumor-adjacent regions tend to have both abnormally large and abnormally small crypt structures. A global average across these values would yield a mean value similar to that derived from normal tissue, but the standard deviation of crypt areas within an image would report this variance in a more representative manner.

The results of image feature quantification were then compared across cohorts: AOM early time point, AOM late time point, AOM-DSS early time point, AOM-DSS late time point, and Control. Nested one-way ANOVA with Tukey’s post-test was used to determine statistically significant differences between the cohorts (JMP Pro 13). For all images, total image acquisition time was approximately 1.5 seconds, with total optical power at the sample limited to prevent photo-damage. Individual images were acquired at 512 x 512 pixels at 14-bit depth, yielding a 522 μm x 522 μm (~0.27 mm2) field of view, at approximately 20 μm from the epithelial surface. Image depth stacks were acquired at consecutive depths from the epithelial surface, in 20 μm steps, from 20 μm to 100 μm below the epithelial surface.

**S5. Fixed image depth results (20 μm)**

The comparison of the means (Fig. S5) and standard deviations (Fig. S6) for each of the image features discussed previously (Fig. S3 and S4) are shown: crypt area, nearest neighbor, circularity, perimeter, crypt count, eccentricity, average centroid distance function, minimum centroid distance function, and maximum centroid distance function. For mean (Fig. S5), the two image features with statistical significance between cohorts were nearest neighbor and crypt count. For standard deviation (Fig. S6), the image features with statistical significance were nearest neighbor, circularity, and eccentricity. They all showed a difference between the Control cohort and one or more of the tumor-bearing cohorts, but this is not outside of what has been shown with confocal laser microendoscopy systems such as the Cellvizio® (which differentiated between colorectal lesions and adjacent normal colorectal epithelial crypt structure in a preliminary clinical trial) [4].

The differences of interest are in the distinction in mean (Fig. S5) of nearest neighbor between the AOM late and AOM-DSS early cohorts, as well as the AOM late and the AOM-DSS late cohorts. For standard deviation (Fig. S6), the difference of interest is also for nearest neighbor, between AOM late and AOM-DSS late cohorts. These results suggest that morphological biomarkers in SHG images of colorectal epithelium could be used to distinguish between spontaneous tumor development (AOM model) and colitis-associated tumor development (AOM-DSS model). While the alternative image features did not show statistical significance in our study, this does not conclusively rule them out as incapable of providing additional information about the morphological differences between cohorts. Further study into these or similar features could prove useful in development of clinical imaging systems and guides for interpreting the acquired images.


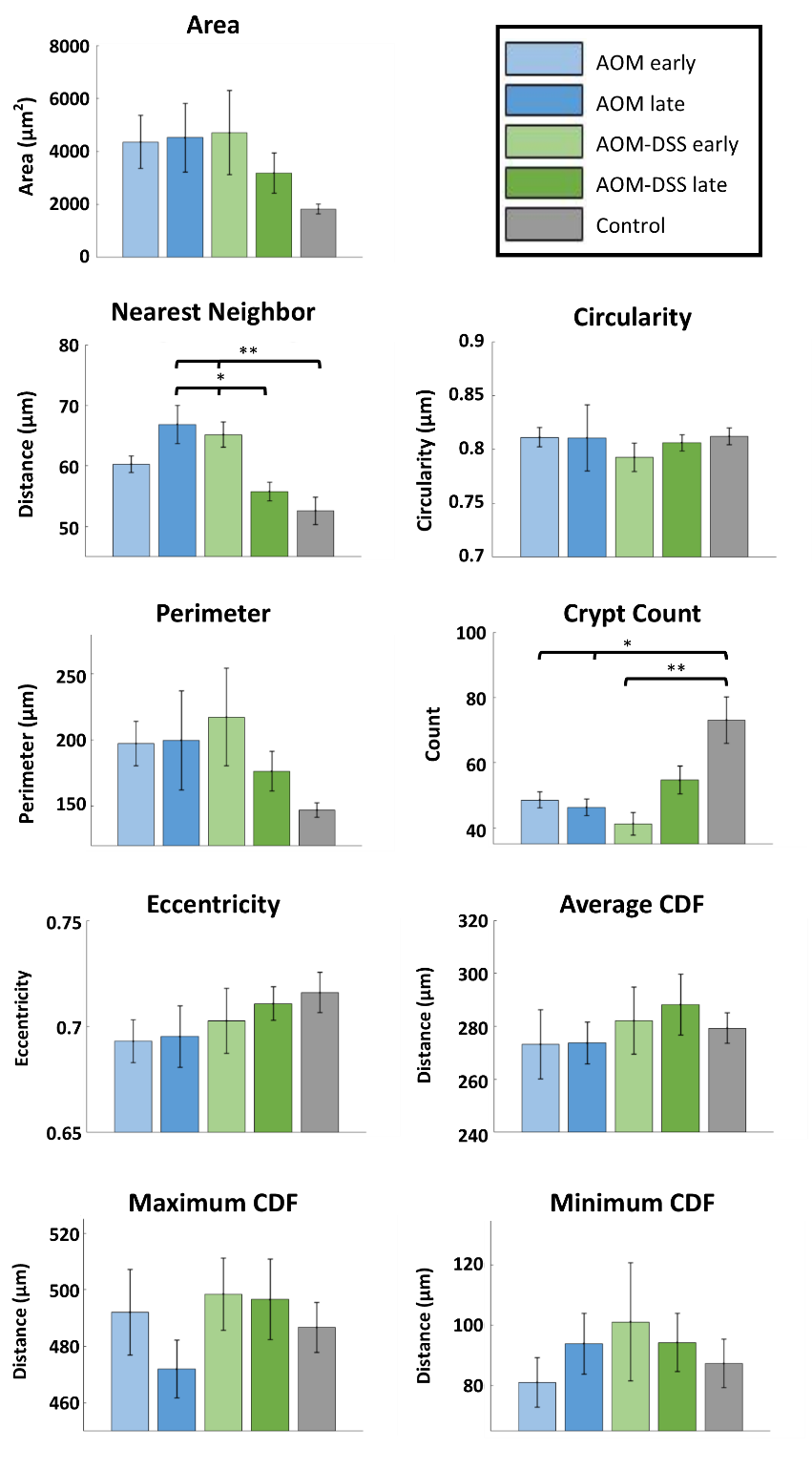


**Figure S5.** Mean values of various image features: crypt count, crypt area, crypt circularity, crypt eccentricity, nearest neighbor, average centroid distance function (CDF). Cohort n values (images): AOM early n=48, AOM late n=23, AOM-DSS early n=45, AOM-DSS late n=24, Control n=55. Error bars are standard error; significance was calculated via a nested one-way ANOVA with Tukey’s HSD post-test. Significance was noted (*) for p values <0.05 and (**) for p values <0.01.


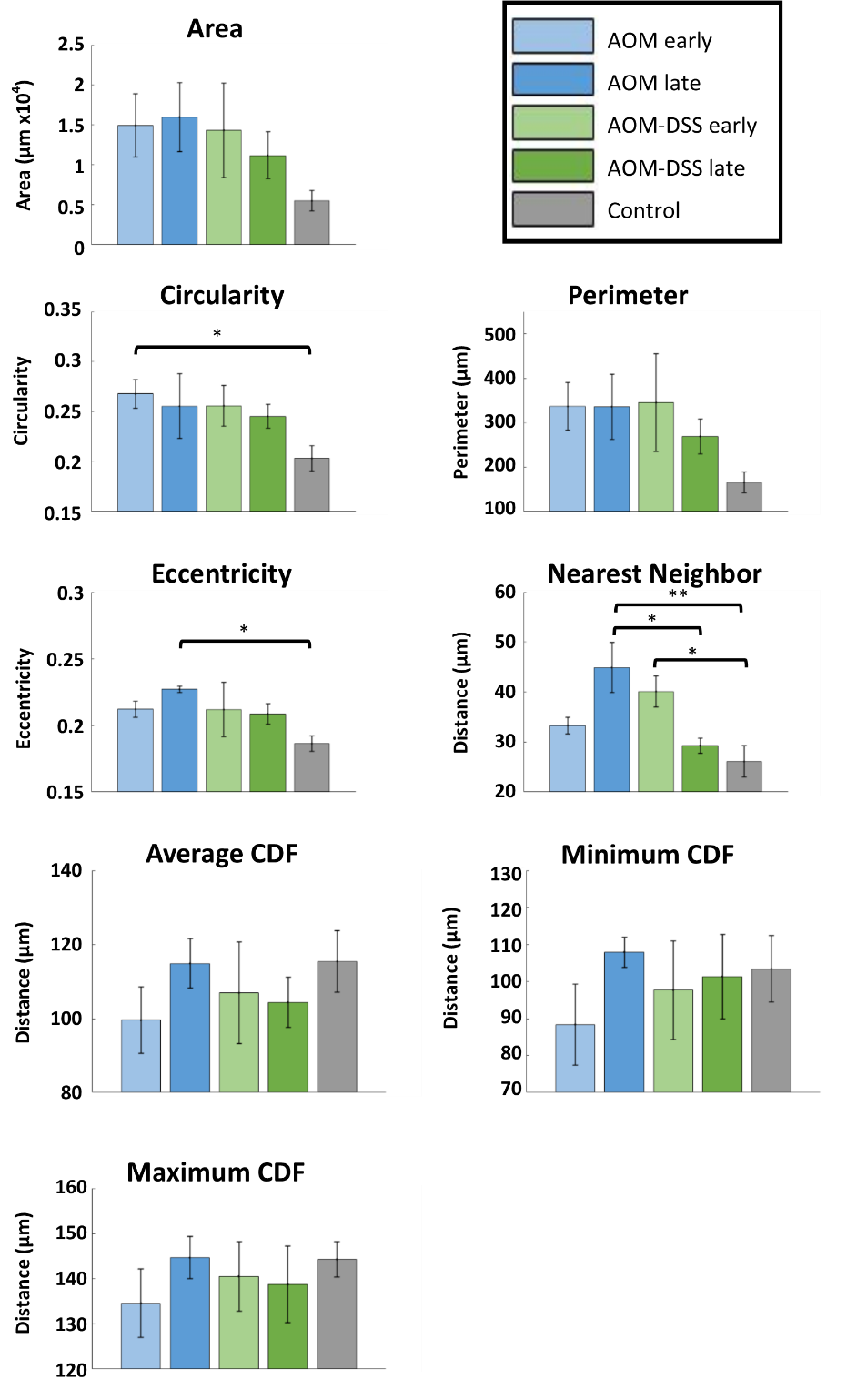


**Figure S6.** Standard deviation values of various image features: crypt count, crypt area, crypt circularity, crypt eccentricity, nearest neighbor, average centroid distance function (CDF). Cohort n values (images): AOM early n=48, AOM late n=23, AOM-DSS early n=45, AOM-DSS late n=24, Control n=55. CDF = centroid distance function. Error bars are standard error; significance was calculated via a nested one-way ANOVA with Tukey’s HSD post-test. Significance was noted (*) for p values <0.05 and (**) for p values <0.01

**S6. Image depth stack results**

The comparison of the means and standard deviations for each of the image features discussed previously (Fig. S3 and S4) were calculated across a fixed depth (20 to 100 μm) and compared across cohorts. The image stacks were also compared within cohorts (fixed groups) across varying acquisition depth for the means and standard deviation of each of the image features. Due to the extensive number of comparisons, as well as due to the majority not showing statistical significance, only the image features with at least one statistically significant difference between two cohorts are shown below. For comparison of means across cohorts (Fig. S7), the image feature with statistical significance between cohorts was nearest neighbor at 40 μm. To compare standard deviation values across cohorts (Fig. S8), the image features with statistical significance were area, perimeter, and nearest neighbor at 40 μm, and circularity at 60 μm. To compare mean values across acquisition depths there are no figures, as no image feature showed statistical significance between cohorts. For comparison of standard deviations across acquisition depths (Fig. S9), the image features with statistical significance were circularity within the AOM late cohort, and circularity and eccentricity within the Control cohort.

Several of the image features, including nearest neighbor, area, perimeter, circularity, and eccentricity showed a difference between the Control cohort and one or more of the tumor-bearing cohorts. While these results corroborate that there are morphological differences between regions of dysplasia and healthy epithelium, they do not show clear usefulness in distinguishing between spontaneous tumor development (AOM model) and colitis-associated tumor development (AOM-DSS model). Though this does not prove them incapable of providing additional information about the morphological differences between cohorts, further study into depth section and/or volumetric analysis of crypt morphology is needed to better understand any differences between spontaneous and colitis-associated tumor development.


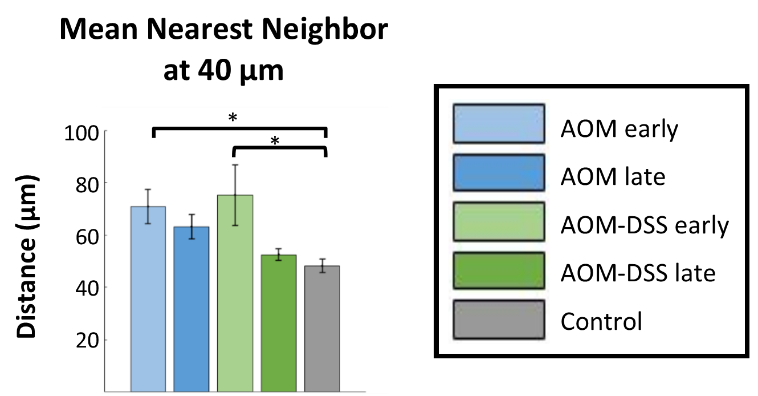


**Figure S7.** Mean values of crypt nearest neighbor at 40 μm of acquisition depth across cohorts. Cohort n values (stacks): AOM early n=8, AOM late n=9, AOM-DSS early n=5, AOM-DSS late n=12, Control n=9. Significance was calculated via two-way ANOVA with Tukey’s post-test. Error bars are standard error Significance was noted (*) for p values <0.05 and (**) for p values <0.01.


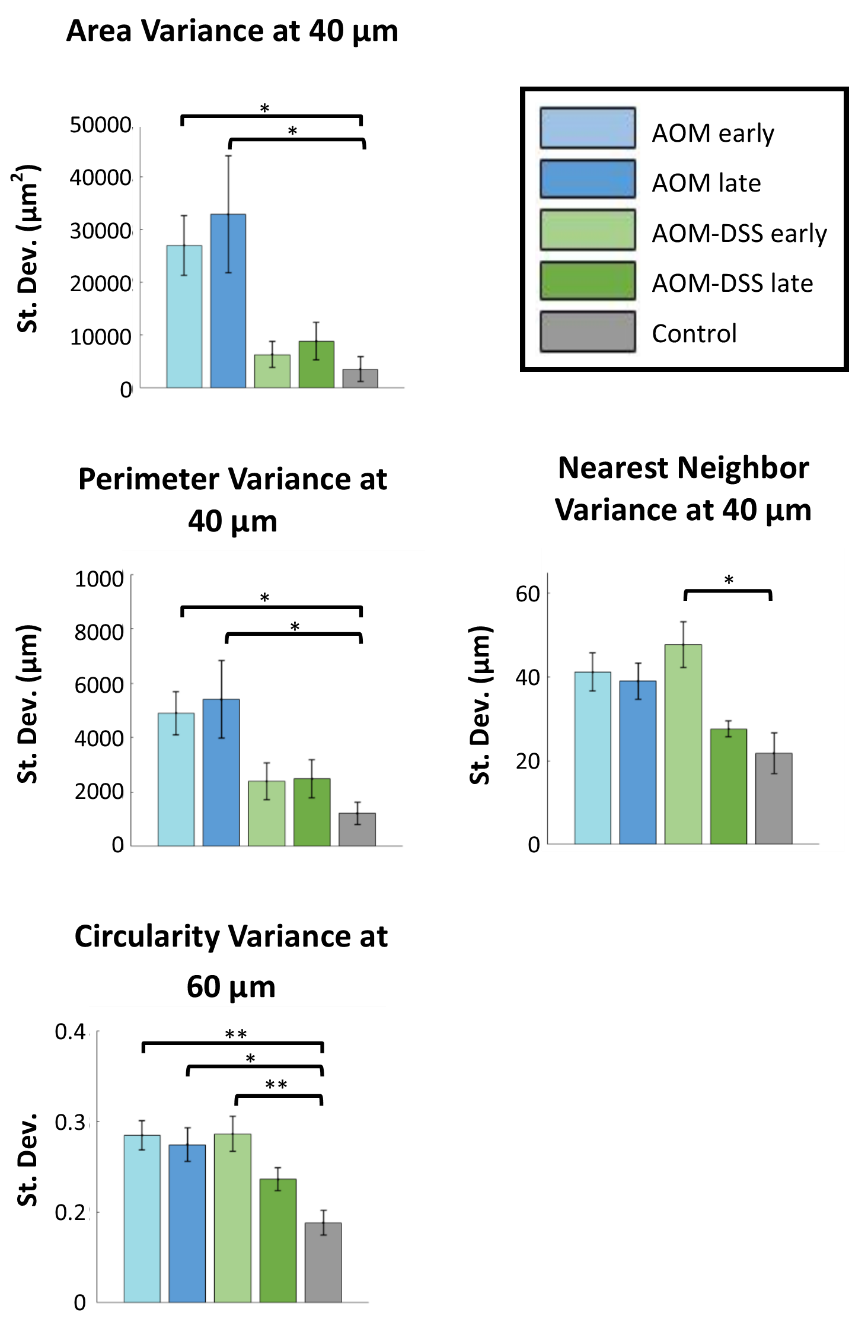


**Figure S8.** Standard deviation values of area, perimeter, and nearest neighbor at 40 μm of acquisition depth across cohorts, and of circularity at 60 μm of acquisition depth across cohorts. Cohort n values (stacks): AOM early n=8, AOM late n=9, AOM-DSS early n=5, AOM-DSS late n=12, Control n=9. Significance was calculated via two-way ANOVA with Tukey’s post-test. Error bars are standard error Significance was noted (*) for p values <0.05 and (**) for p values <0.01.


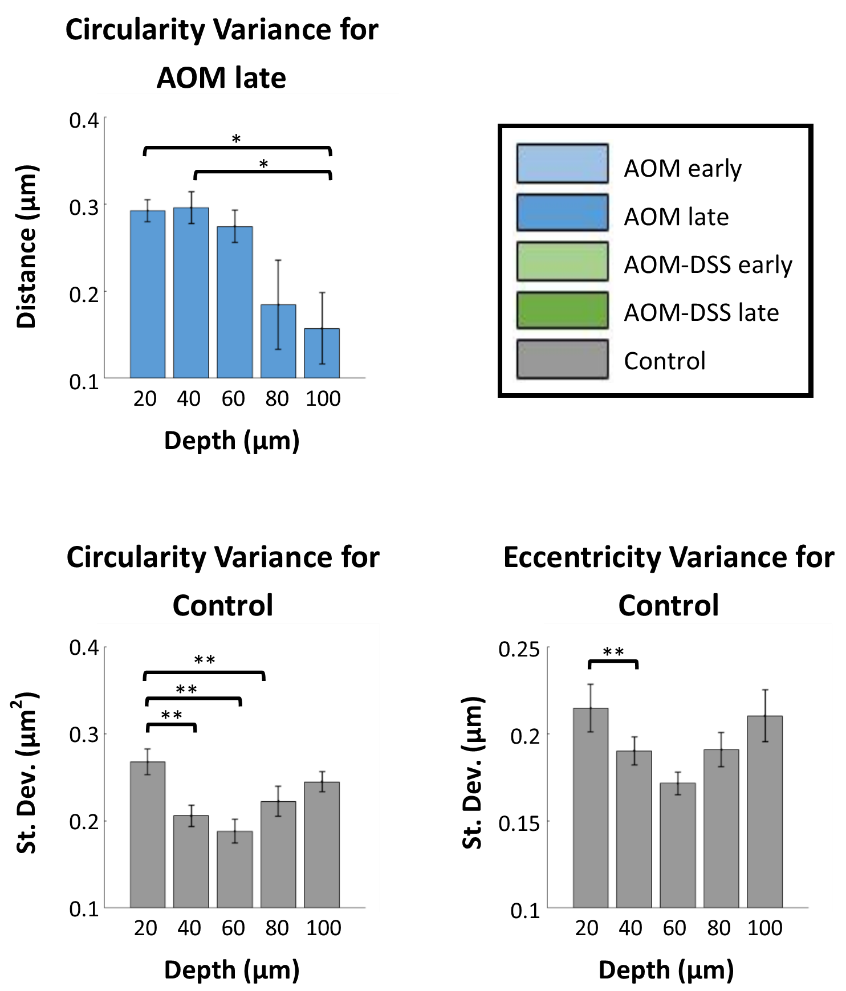


**Figure S9.** Standard deviation values of crypt circularity of AOM late cohort across acquisition depths, and of crypt circularity and eccentricity of Control cohort across acquisition depth. Cohort n values (stacks): AOM late n=9, Control n=9. Significance was calculated via two-way ANOVA with Tukey’s post-test. Error bars are standard error Significance was noted (*) for p values <0.05 and (**) for p values <0.01.

**References**

1. Prieto SP, Lai KK, Laryea JA, Mizell JS, Muldoon TJ: **Quantitative analysis of ex vivo colorectal epithelium using an automated feature extraction algorithm for microendoscopy image data.** Journal of Medical Imaging 2016, **3**(2):024502.

2. Prieto SP, Lai KK, Laryea JA, Mizell JS, Mustain WC, Muldoon TJ: **Fluorescein as a topical fluorescent contrast agent for quantitative microendoscopic inspection of colorectal epithelium.** Biomedical optics express 2017, **8**(4):2324-2338.

3. Rivera DR, Brown CM, Ouzounov DG, Pavlova I, Kobat D, Webb WW, Xu C: **Compact and flexible raster scanning multiphoton endoscope capable of imaging unstained tissue.** Proc Natl Acad Sci U S A 2011, **108**(43):17598-17603.

4. De Palma GD, Staibano S, Siciliano S, Maione F, Siano M, Esposito D, Persico G: **In-vivo characterization of DALM in ulcerative colitis with high-resolution probe-based confocal laser endomicroscopy.** World J Gastroenterol 2011, **17**(5):677-680.
